# SFPD: Simultaneous Face and Person Detection in Real-Time for Human–Robot Interaction

**DOI:** 10.3390/s21175918

**Published:** 2021-09-02

**Authors:** Marc-André Fiedler, Philipp Werner, Aly Khalifa, Ayoub Al-Hamadi

**Affiliations:** Neuro-Information Technology Group, Otto von Guericke University Magdeburg, 39106 Magdeburg, Germany; philipp.werner@ovgu.de (P.W.); aly.khalifa@ovgu.de (A.K.); ayoub.al-hamadi@ovgu.de (A.A.-H.)

**Keywords:** face detection, person detection, multi-task learning, real-time detection

## Abstract

Face and person detection are important tasks in computer vision, as they represent the first component in many recognition systems, such as face recognition, facial expression analysis, body pose estimation, face attribute detection, or human action recognition. Thereby, their detection rate and runtime are crucial for the performance of the overall system. In this paper, we combine both face and person detection in one framework with the goal of reaching a detection performance that is competitive to the state of the art of lightweight object-specific networks while maintaining real-time processing speed for both detection tasks together. In order to combine face and person detection in one network, we applied multi-task learning. The difficulty lies in the fact that no datasets are available that contain both face as well as person annotations. Since we did not have the resources to manually annotate the datasets, as it is very time-consuming and automatic generation of ground truths results in annotations of poor quality, we solve this issue algorithmically by applying a special training procedure and network architecture without the need of creating new labels. Our newly developed method called Simultaneous Face and Person Detection (SFPD) is able to detect persons and faces with 40 frames per second. Because of this good trade-off between detection performance and inference time, SFPD represents a useful and valuable real-time framework especially for a multitude of real-world applications such as, e.g., human–robot interaction.

## 1. Introduction

The detection of face and person bounding boxes from images is very important for a variety of applications. For example, they can be used in the field of human–computer interaction (HCI) to detect possible interaction partners, in autonomous driving to perceive road users such as pedestrians, or in mobile robot navigation to identify moving obstacles. Furthermore, they are the first component for a large number of recognition systems in many applications, such as face recognition [1], facial expression analysis [2,3], body pose estimation [4], face attribute detection [5], human action recognition [6] and others. In such systems, face and/or person detection are often a prerequisite for the following processing steps; so, their detection rate is crucial for the performance of the overall system. Through deep learning, the results in the area of object detection have been greatly improved. However, many state-of-the-art approaches that use deep neural networks require very heavy computation so that inference does not run in real-time on a conventional graphics processing unit (GPU), which severely limits their suitability for many real-world applications that require high framerates.

Our application, for which we combined face and person detection, lies in the area of autonomous robotic systems. The robot must be able to detect persons with their faces in real-time, especially, in close range to the system with only limited computational capacity in order to perform HCI. However, the use of our framework is not limited to this field of application and is useful for many more real-world applications.

A major difficulty for the integration of the two tasks, face and person detection, in a single neural network is the fact that publicly available databases contain only ground truths for one of the two tasks. To the best of our knowledge, there is no extensive dataset containing coordinates of face as well as person bounding boxes. To perform the two tasks simultaneously within the same convolutional neural network (CNN), it is trained using multi-task learning (MTL). The distinctive characteristic of our training procedure lies in the fact that we train our network in a single continuous process simultaneously on both databases for the tasks of face and person detection, although ground truths are missing for one of the two classes in each database. Thereby, we are able to handle this circumstance without the need of generating new labels, since the manual generation of annotations is very time-consuming and the automatic generation only results in annotations of poor quality. To our knowledge, such a training process has not been presented in the research community so far.

In this work, we propose an MTL framework for simultaneous detection of faces and persons, which is able to process 40 frames per second (fps) and is therefore more than real-time capable. This makes it possible to add further downstream recognition tasks to the framework and still maintain its real-time runtime. Thus, the algorithm is very interesting for real-world applications. The results achieved on the WIDER Face [7] and Pascal VOC [8,9] datasets can compete with other lightweight state-of-the-art methods. In addition, our framework is completely end-to-end trainable, without pre-training individual network parts, splitting up the training process, freezing single network layers or creating additional annotations for one database, as it is mostly the case with other MTL networks.

The main contributions of our work can be summarized as follows:

1. We propose a new CNN for Simultaneous Face and Person Detection (SFPD) in real-time, which is completely end-to-end trainable using MTL with two datasets, each containing the ground truths for one of the two detection tasks;
2. A new network architecture was developed which consists of a joint backbone with shared feature maps and separate detection layers for each task;
3. A multi-task loss was designed which allows to generate loss values throughout the whole training process despite missing ground truth labels in the training datasets;
4. Comprehensive experimental validation was performed by comparing the detection performance and inference runtime of multiple algorithms.

Our paper is structured in the following way: In Section 2, related work on general object detection, face detection, and multi-task learning is reviewed. In Section 3, our method is presented in detail with regard to the used network architecture and loss function. In Section 4, the experiments and their results are reported providing details on the training procedure and the datasets used. Finally, in Section 5, conclusions are drawn.

## 2. Related Work

There are three major research areas related to our work: general object detection, face detection and multi-task learning. This section gives a brief summary about these areas.

### 2.1. Object Detection

The general goal of object detection is to localize the borders of a wide range of objects inside an image. These object boundaries are described using bounding boxes and are intended to fit as closely as possible to the object shapes. Additionally, a class label is predicted as output for each detected object. It is possible that the image contains multiple objects. The difference to image classification lies in the fact that in classification there is only one object in the image whose class label is predicted as output, but the bounding box is not localized.

Especially due to the developments in the field of deep CNNs, the performance of detection tasks could be increased significantly in recent years. This can be attributed to the large amount of annotated training data, as well as to the availability of more powerful GPUs, enabling the training of increasingly deeper and more complex network architectures. However, still the most accurate modern neural networks do not operate in real-time and require large number of GPUs for training with a large mini-batch size [10]. Thus, these methods often cannot be applied for real-world applications with specific requirements regarding the runtime, hardware, energy consumption, etc.

Modern detection frameworks usually consist of two parts: A backbone for obtaining the features, which is often pre-trained on ImageNet [11], and a head for predicting the object classes and bounding box coordinates. Thereby, the head parts can be categorized into single-stage and two-stage detectors.

Two-stage detectors initially generate a large amount of generic object proposals. For this purpose, they use external algorithms, such as Selective Search [12], Egde Boxes [13] or Adobe Boxes [14]. In more recent approaches, the generation of object proposals is integrated into the network structure by using a region proposal network making the framework end-to-end trainable. In the next step, each region proposal is classified, whether it contains an object or not using a CNN. The first two-stage object detection algorithm was R-CNN [15], upon which newer variants, such as Fast R-CNN [16], Faster R-CNN [17], R-FCN [18], Mask R-CNN [19] and Libra R-CNN [20] are based on. Although the two-stage detectors have the capability to achieve the best detection accuracy, they are rarely used in practice because of their limited suitability for real-time systems. This is primarily due to the generation of region proposals, which is a computationally intensive process and the main bottleneck for reaching a real-time detection framework.

Single-stage detectors, often also called single-shot detectors, directly compute object confidence scores and bounding box coordinates for a given input image without generating region proposals. For this purpose, a fixed set of anchor boxes with different aspect ratios and scales is applied to all image components in order to be able to immediately predict the confidence scores. This greatly improves the detection speed and enables real-time detection, while reducing the detection accuracy [21]. Due to the better processing speed, the single-stage detectors are used in practice much more often. To ensure detection of differently scaled objects in a single forward pass through the network, they utilize the built-in pyramid structure of CNNs. Feature maps from different stages of layers with various sizes are collected and pooled, allowing the network to perform direct object classification and regression of bounding boxes for several scales of objects. The most representative models for single-stage object detectors are the versions of YOLO [10,22,23,24], SSD [25] and RetinaNet [26]. In recent years, more approaches have been introduced: EfficientDet [27] is a scalable object detection framework where it is easily possible to change the backbone in order to optimize accuracy and efficiency of the network. With FCOS [28] and FoveaBox [29], two anchor-free frameworks have been introduced. Their advantage lies in the fact that complicated computations related to anchor boxes such as overlaps during training are avoided by eliminating the predefined set of anchors. Instead, pixel-wise classification is applied to the feature map outputs of the backbone, similar to semantic segmentation, for detecting the objects.

The recognition task of person detection is mainly handled within the general object detection, because most object recognition datasets have persons annotated as one of their object categories. Therefore, most general object detection frameworks perform the detection of persons besides further object classes.

### 2.2. Face Detection

Face detection is a specialization of general object detection, which focuses on the detection of human faces. Many algorithms for face detection have been derived from methods for general object detection.

Before deep learning became the standard in object and face detection, manually acquired features were used to accomplish the detection tasks. One of the most popular algorithms for face detection was developed by Viola and Jones [30]. It utilizes Haar-Like features and AdaBoost [31] learning to train cascaded classifiers, which achieve good performance in real-time speed. Besides Viola and Jones, the deformable parts model (DPM) [32] has been proposed in the literature [33,34,35] for face detection using histogram of oriented gradient (HOG) [36] features, which are robust to partial occlusion and define a face as a collection of its parts. The main problem for the usage of Haar-Like and HOG features in unconstrained face detection lies in their inability to capture facial information at different resolution, viewpoint, illumination, expression, skin color, occlusions and cosmetic conditions [37].

To overcome these limitations, various deep learning-based face detection models have been introduced in the literature. One of the first CNN-based face detection algorithms is Cascade-CNN [38]. It uses an image pyramid to detect differently scaled faces. Then, it merges the individual faces detected from pyramid structure for the whole image using non-maximum suppression (NMS) [39], discarding strongly overlapping bounding boxes. A similar cascade is used by Multi-scale Cascade CNN [7] and by MTCNN [40], while MTCNN additionally captures five facial landmarks for improved face detection.

In recent years, many more algorithms have been introduced: Face R-FCN [41] is built on the R-FCN [18] framework and is optimized for face detection. To improve detection accuracy, they exploit position-sensitive average pooling, multi-scale training and testing as well as on-line hard example mining. S${}^{3}$FD [42] consists of a scale-invariant network with a new anchor matching strategy for improved recall rate on tiny faces. In order to increase performance in particular for partially occluded faces, the specially developed approach FAN [43] uses anchor-level attention maps. In PyramidBox [44], the authors applied context modules on feature pyramids to enlarge the receptive field for better observation of context information. ScaleFace [45] is able to handle an extremely wide range of scales using a specialized set of deep CNNs with different structures. The challenging problem of simultaneous dense localization and alignment of faces of arbitrary scales in images is addressed in RetinaFace [46] through adding a self-supervised mesh decoder branch for additional prediction of pixel-wise 3D shape information. DSFD [47] proposes a novel feature enhance module and an enhanced anchor matching strategy for obtaining more discriminability and better initialization for the regressor. DBCFace [48] is an anchor-free face detector that generates binary segmentation masks indicating for each pixel whether it belongs to a face or not.

Due to this multitude of developments, the performance in the field of face detection has been enhanced significantly. However, the performance of the algorithms is also strongly correlated to the required computation time, which is the reason why almost none of the previous mentioned deep learning approaches are able to run in real-time on a conventional GPU, e.g., PyramidBox [44] only achieves 3 fps on an NVIDIA Titan RTX (Nvidia Corporation, Santa Clara, CA, USA) and ScaleFace [45] only 4 fps on an NVIDIA Titan X. One approach that combines good results with real-time runtime is YOLO-face [49]. The method was developed based on YOLOv3 [24] and reaches 38 fps on an NVIDIA GeForce GTX 1080 Ti.

### 2.3. Multi-Task Learning (MTL)

MTL describes the simultaneous learning of multiple tasks at the same time, whereby several output targets are generated for one input target [50]. MTL for machine learning was first introduced by Caruana [51] in 1998. However, before deep learning algorithms were extensively deployed, it was highly limited to just a few use cases as the required features strongly differed. With the upcoming trend of using CNNs for computer vision tasks and the rejection of hand-crafted features, the fields of application for MTL could be extended considerably.

Several MTL frameworks were presented such as: DAGER [52] for age, gender and emotion recognition; HyperFace [53] for face detection, pose estimation, landmark localization and gender recognition; or All-In-One [54] for face detection, landmark localization, face recognition, 3D head pose estimation, smile detection, facial age estimation and gender classification. Additionally, Levi and Hassner [55] proposed a CNN for age and gender estimation, Zhang et al. [56] optimized facial landmark localization with facial attribute inference and head pose estimation, and Gkioxari et al. [57] trained a CNN for person pose estimation and action detection.

Chen et al. [58] proposed to combine face detection and alignment in one framework, because they observed that aligned face shapes provide better features for face detection. Furthermore, Saxen et al. [59] proved that a CNN can detect faces more easily by adding face orientation as a training target. Inspired by these approaches, various methods for face detection were developed, which incorporated the prediction of additional facial features into the network for improved performance: MTCNN [40] and RetinaFace [46] predict five ancillary face landmarks, He et al. [60] predict plenty facial attributes and Wu et al. [61] predict the head pose.

The advantage of having an MTL network, instead of constructing independent CNNs for each task, is to profit from the inherent correlation between the related tasks and thereby to enhance each others performance [61]. By sharing the feature maps for the different detection layers, the generalization capability of the features improves and they can adapt more effectively to the complete set of recognition domains. This enhances both learning efficiency and prediction accuracy [62]. In addition, the shared use of several CNN layers reduces the computational time, which helps realizing a real-time system for simultaneous execution of multiple tasks.

## 3. Method

This section introduces our new method for simultaneous face and person detection, called SFPD, in detail. The basic design of our SFPD algorithm is inspired by the SSD [25] framework. The layout of the network architecture and the applied loss function are explained in the following subsections.

The novelty of our training procedure and network architecture lies in the fact that it is trained end-to-end on two datasets which are both only partially annotated and therefore only contain labels for one of the two target object classes (faces and persons). We solve this problem algorithmically without an additional generation of new ground truths, since we do not have the resources to generate new labels manually as it is very time-consuming and automatically generated labels are of worse quality. For this reason, the training process alternates between batches with face and batches with person annotations. Details about the training data can be found in Section 4.1, details about the training procedure in Section 4.2.

### 3.1. Network Architecture

**Figure 1:**
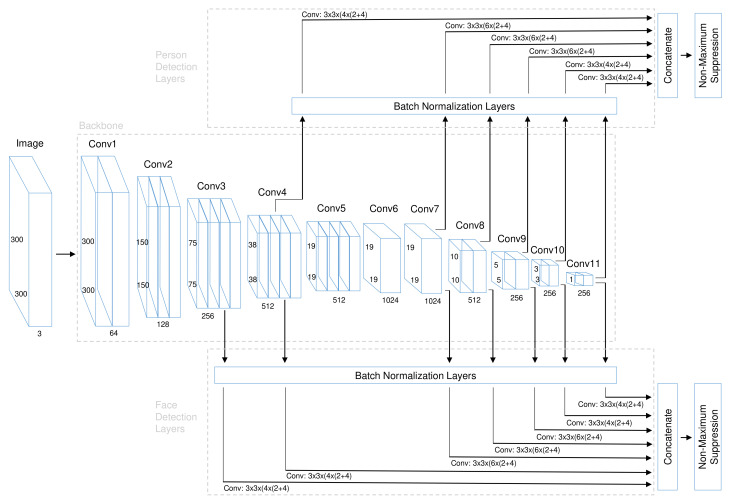
Network architecture of SFPD. It consists of a shared backbone and separate detection layers for face and person detection.

Our SFPD algorithm belongs to the group of single-stage object detectors (see Section 2.1) and is a feed-forward CNN which uses predefined anchor boxes to output bounding box coordinates and confidence scores for the respectively targeted class. The network architecture of SFPD consists of two parts: A joint backbone with shared feature maps and separate detection layers for the single detection tasks. The detailed structure is illustrated in Figure 1.

The backbone generates base features and is shared by the two detection layer branches for faces and persons. This becomes possible because the first layers extract very rough features such as contours and edges. The middle and back layers of the backbone already exhibit specific task-related features, which however gain better generalization ability through training on two related detection tasks. The first part of our backbone consists of the VGG-16 [63] network. Each of these convolutional blocks (conv1–conv5) consists of a series connection of one or more convolutional layers with rectified linear unit (ReLU) activation function and a kernel size of 3 × 3 followed by a max pooling layer with 2 × 2 kernel. All weights were pre-initialized with values trained on ImageNet [11]. The ReLU activation is able to increase the overall non-linear fitting ability of the CNN. Similar as in SSD [25], the fully connected layers fc6 and fc7 are replaced by convolutional layers (conv6 and conv7) with 1024 filters each, fully connected layer fc8 is removed and four additional convolutional layer blocks (conv8 to conv11) with two convolutional layers each and successive kernel sizes 1 × 1 and 3 × 3 are added at the end of the VGG-16 [63] network. The layers of the first additional block have 256 and 512 filters, those of the following three ones first 128 and then 256 filters. This results in a feature map size of 1 × 1 at the end of the backbone for input images with 300 × 300 pixels. The advantage of the 1 × 1 convolution lies in the fact that it performs the dimension reduction of the feature map without significantly increasing the number of parameters. The newly added convolutional layers are initialized by the Xavier [64] method.

The detection layers generate as output the bounding box coordinates and percentage class confidence scores for each detection task. Therefore, each detection layer consists of two head layers, one for bounding box regression and one for class prediction. In order to be able to detect persons and faces of different scales in one pass through the CNN without generating image pyramids, features must be tapped at different levels of the backbone. This is possible because the layers of the backbone are progressively decreasing in size. The taps for the detection layers are located after layer conv4-3 and conv7 (formerly fc7) as well as at the end of each newly added block following the VGG-16 [63] network. In order to be able to detect more smaller faces, a seventh tap after conv3-3 is added to the branch for the face detection layers. The detection layer for each tap consists of a batch normalization layer followed by two parallel convolutional layers corresponding to the two heads. Afterwards, all detection layer feature maps of a branch are concatenated in order to aggregate the multi-scale detections. The entire CNN is composed of 24, 453, 160 parameters in total from which 24, 451, 112 are trainable.

The anchor boxes are very important hyper-parameters and crucial for the later detection performance. A set of anchor boxes with different sizes and aspect ratios is assigned to each detection layer feature map allowing to cover suitable boxes for a large range of faces and persons that may appear in the images. Usually, the height of faces and persons in images is greater than the width. Therefore, besides square anchor boxes, additional ones with aspect ratios of one half and one third are applied. However, the test data showed exceptions to this assumption. For that reason the flipped anchor boxes with aspect ratios two and three were also added. The anchor box sizes were adopted from the original SSD300 [25] implementation.

The SFPD network outputs a fixed-sized set of bounding boxes and their respective confidence scores for the presence of a face or person. During inference, the final detections must be generated out of these. Most boxes can already be sorted out by the confidence threshold. The confidence threshold plays an important role, because if it is set too high, correct detections are rejected and if it is set too low, many false positives remain in the results. Depending on the layer where the bounding boxes are tapped, we use different confidence scores because it has been observed that especially for small objects, it is often difficult to achieve a sufficiently high score. Therefore, the bounding boxes from the first two person and the first three face detection layers receive a confidence threshold of 0.1, the next two of 0.2 and the last two of 0.3. To avoid multiplicate detection of the same object, NMS is used. Boxes with an intersection over union (IoU) of more than 0.5 are rejected and a maximum of 300 detections is kept per image. The decision is based on the highest confidence score.

### 3.2. Loss Function

During the training of our SFPD network a loss function consisting of multiple parts is optimized. For each detection branch, a loss is calculated consisting of a confidence loss ($L_{conf}$) for the confidence scores and a regression loss ($L_{reg}$) for the bounding box coordinates.

Since the two detection layers decide for each anchor box, if it contains a person or a face (depending on the branch) or if the box is classified as background, these are both binary decision problems. For this reason, we use the binary cross-entropy loss for $L_{conf}$:

(1) $$L_{conf}=-\frac{1}{N}\sum_{i=1}^{N}y_{i}\;log\left({\hat{y}}_{i}\right)+\left(1-y_{i}\right)\;log\left(1-{\hat{y}}_{i}\right)$$

where ${\hat{y}}_{i}$ is the model output for the *i*-th anchor box, $y_{i}$ is the corresponding target value and *N* is the number of anchor boxes.

We use the generalized intersection over union (GIoU) [65] loss for $L_{reg}$:

(2) $$L_{reg}=\frac{1}{M}\sum_{j=1}^{M}1-IoU_{j}+\frac{A_{j}^{c}-U_{j}}{A_{j}^{c}}$$

where $IoU_{j}$ is the intersection over union between the predicted and ground truth bounding box for the *j*-th anchor box remaining after hard negative mining, $A_{j}^{c}$ the smallest enclosing area and $U_{j}$ the union area between the two bounding boxes. *M* is the total number of remaining anchor boxes after hard negative mining. GIoU was chosen for the regression loss because it is superior to other loss functions in the regression of 2D bounding boxes [65].

The losses for face ($L_{face}$) and person ($L_{person}$) detection are calculated from the respective $L_{conf}$ and $L_{reg}$:

(3) $$L_{face}=L_{conf\_face}+2\times L_{reg\_face}$$

(4) $$L_{person}=L_{conf\_person}+2\times L_{reg\_person}$$

Thereby, the regression loss is weighted twice as high as the confidence loss. The weight was chosen empirically and resulted in an improved optimization during training. Learning the binary classification proved to be uncritical even with weaker weighting.

To realize a complete end-to-end trainable framework for both detection tasks, a total loss function is required. This total loss *L* is composed of the loss functions for faces and persons:

(5) $$L=3\alpha \times L_{face}+\beta \times L_{person}$$

Our training process alternates between batches of face and batches of person samples, which come from different databases. During training a batch with face annotations α=1 and β=0 are set, during a batch with person annotations α=0 and β=1 are set. This ensures a steady calculation of the loss during the whole training process, despite the fact that one of the two ground truths is missing for the input images. The face loss is triple-weighted compared to the person loss because it has been observed that otherwise the network optimizes itself strongly in the direction of person detection and neglects face detection to a large extent.

By applying this loss function, a network could be designed which is able to detect faces and persons simultaneously. The framework is completely end-to-end trainable, although the available datasets have either face or person labels, but no dataset has both. Details about the exact training procedure can be found in Section 4.2.

## 4. Experiments and Results

This section describes the experiments and their results in detail. First, the datasets used for training and testing our SFPD network are introduced and, then, the training procedure is precisely specified. Afterwards, the achieved results are presented and discussed. Finally, the limitations of our new algorithm are pointed out.

### 4.1. Datasets

Training a CNN for simultaneous detection of faces and persons in images is not a straightforward task, as extensive and publicly available datasets, which contain face as well as person bounding box annotations, do not exist in the research community. In order to train such a network, partially annotated datasets have to be used.

For training and testing the face detection task, we utilize the WIDER Face [7] dataset. It is currently the most popular and commonly used dataset in face detection. Besides, it is very challenging due to the high variability in scale, pose, expression and occlusion of the faces pictured in its images. For training, we apply the WIDER train set with 12,880 images and, for testing, the WIDER validation set with 3226 images. The sets are divided into the three categories “easy”, “medium” and “hard” according to their level of difficulty for detection.

The task of person detection is trained and tested using the Pascal VOC datasets [8,9] from 2007 and 2012. The two datasets contain annotations for 20 different object classes, however, we are only interested in the person annotations. For this reason, all images without person annotations are sorted out. In addition, annotations of other object classes are ignored during training. This results in 2095 remaining images for the VOC 2007 trainval set and 9583 for the VOC 2012 trainval set, which are used for training the SFPD network. In total, this results in 11,678 training images with person annotations, which leads to a relatively balanced number of training images between the two detection tasks compared to 12,880 for WIDER train. No negative samples (without faces and without persons) were used in the training as the test performance showed no need for this, since no false positives were detected on images without objects. The same procedure for rejecting images is applied to the person test sets. This leaves 2097 images in the VOC 2007 test set and 5138 in the VOC 2012 test set for testing the person detection of our SFPD network.

### 4.2. Training Procedure

The SFPD algorithm has been trained on partially annotated databases because there is a lack of datasets with person as well as face annotations. Therefore, the training procedure is slightly more complex compared to other CNNs.

First, the input images are loaded and scaled to a size of 300 × 300 pixels. Thereby, a batch size of 32 is used. Each batch contains only images with either face or person annotations. Batches with mixed images from both detection tasks do not occur in our training process. The images within the batches are randomly selected from the dataset. Whether a face or person batch is loaded, is determined by the probability calculated as the ratio of the total number of face to person batches. The training epoch ends once all batches of the three training datasets have been loaded.

To increase the generalization capability of the network, various data augmentation techniques are applied to the input images. The images are flipped horizontally with a probability of 0.5 and vertically with 0.1. Furthermore, every third image is rotated in the range of −30 to 30 degrees. Since it is difficult for the network to detect small objects, additional training data are generated. Therefore, the images are effectively downscaled to create smaller faces and persons. For this purpose, every third image is expanded by a black area, which extends the original image size by a random factor between one and four. The aspect ratio remains unchanged. Additionally, some photometric distortions are applied on the input images, such as adjusting the brightness, contrast, saturation and hue.

During training, the anchor boxes have to be matched to the ground truth coordinates. Each anchor box above an IoU threshold of 0.5 is classified as positive. This simplifies the learning problem because the network should not only find the one anchor box with the highest IoU overlap, but should also predict high confidence scores for multiple appropriate anchor boxes. During inference, these multiple detections are sorted out using NMS. Since the number of negative anchor boxes greatly exceeds the number of positive ones at training time, hard negative mining is performed to compensate for this imbalance. The negative classified anchor boxes with the highest confidence scores are selected to obtain a ratio of 3:1 between negative and positive training samples.

All training is performed on an NVIDIA GeForce RTX 2080 Ti GPU. The total number of training epochs is 130. We start with a learning rate of $10^{-4}$ which increases by factor 10 after the first ten epochs. By starting the training directly with a higher learning rate, an unstable behavior could be observed. Therefore, it is increased after the weights of the network have reached a more stable state. After 80 and 100 epochs, the learning rate is then reduced by a factor of 0.1 each time. As optimizer, we utilize stochastic gradient descent (SGD) with a momentum of 0.9.

### 4.3. Evaluation Results and Discussion

The evaluation of our SFPD network, which is able to detect faces and persons simultaneously, was conducted on task-specific datasets for each detection target.

**Figure 2:**
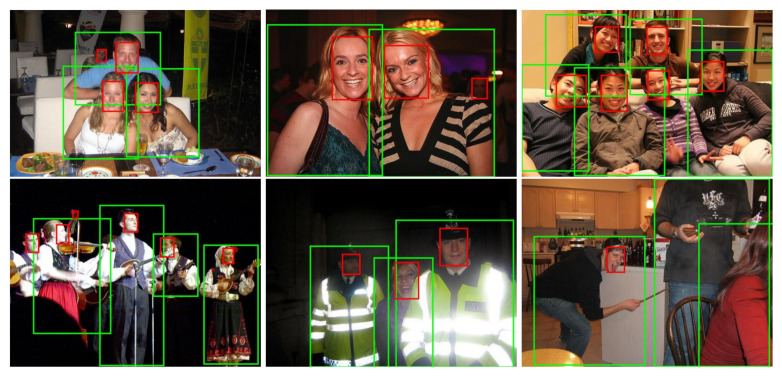
Example detections of SFPD on the Pascal VOC [8,9] test sets 2007 and 2012: Red bounding boxes indicate detected faces; green bounding boxes detected persons.

To evaluate the person detection, the Pascal VOC [8,9] “person” subsets of 2007 and 2012 were chosen. The results obtained with our SFPD method and other algorithms are presented in Table 1. Sample images from the databases with SFPD detections are shown in Figure 2. Our SFPD method outperforms the comparison algorithms Fast R-CNN [16], Faster R-CNN [17], SSD [25] and the first two versions of YOLO [22,23], which are among the most commonly used object detection frameworks. SFPD has one of the fastest computation times considering that both faces and persons are detected in 40 fps and the Titan X, Titan V and RTX 2080 Ti are GPUs with comparable technical specifications. The average precision score was improved by about two percent compared to SSD [25] with unchanged input image size of 300 × 300 on both datasets. Compared to EfficientDet-D2 [27], SFPD shows similar performance results but detects faces additionally to persons. However, the comparison is not quite fair since EfficientDet-D2 [27] was trained on the significantly larger MS COCO dataset. The same applies to EfficientDet-D3 [27], which achieves improved detection results but can only process 27 fps. SSD512 [25], RetinaNet [26] and FoveaBox [29] show slightly higher results of less than two percent, however, they are not even half as fast as SFPD and only manage to generate person bounding boxes in this amount of time.

**Figure 3:**
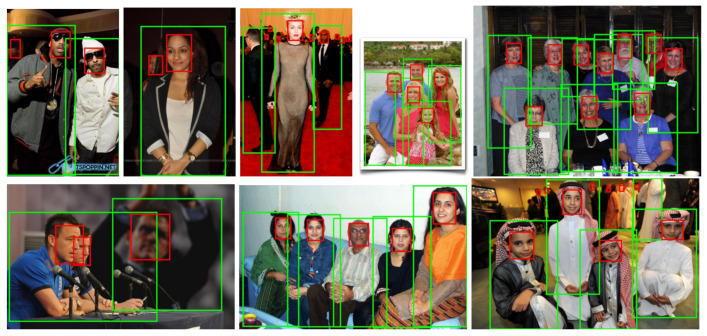
Example detections of SFPD on the WIDER Face [7] validation set: Red bounding boxes indicate detected faces; green bounding boxes detected persons.

**Figure 4:**
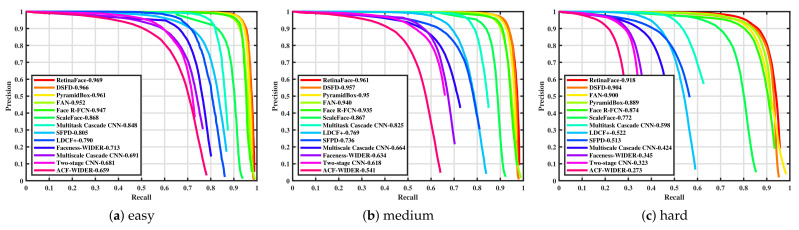
Precision–recall curves of our SFPD network and other detectors on the WIDER Face validation set: (**a**) easy, (**b**) medium and (**c**) hard.

Face detection was tested on the three WIDER Face [7] validation subsets. The results for several of these detection algorithms are listed in Table 2. Furthermore, images with sample detections of SFPD are shown in Figure 3. Corresponding precision–recall curves are outlined in Figure 4. SFPD was compared with a variety of algorithms. The results show that there is only a small number of algorithms that achieve satisfying performance combined with real-time runtime on this dataset. All approaches with average precision values above 90 percent are not able to be executed in real-time. DSFD [47] with a ResNet50 architecture represents an exception and is capable of running almost in real-time with 22 fps on a high-end Tesla P40 GPU. All other methods at the top of the results list are far below this runtime. This shows that face detection is a complex and computing intensive computer vision task. The two implementations of YOLO-face [49] indicate the best trade-off between performance and runtime achieving 89.9 percent at 38 fps and 82.5 percent at 45 fps on the “easy” subset. Our SFPD ranks just below them in terms of performance. The average precision score is between one and four percent worse on each of the three subsets than YOLO-face [49] with darknet-53 architecture. The framerates are in similar range, but it has to be mentioned that SFPD additionally detects persons in the same amount of time and no additional CNN is needed for this purpose.

**Figure 5:**
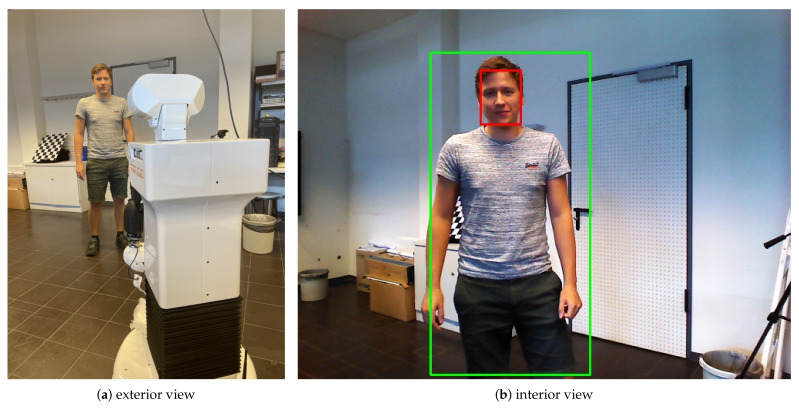
Our proposed SFPD network implemented on a mobile robot system for human–robot interaction in a demo application: (**a**) exterior view and (**b**) interior view of the robot.

While the performance gap between the “easy” and “medium” subset is not very big, SFPD has to deal with a drop of more than 22 percent between “medium” and “hard”. This can be explained by the fact that the “hard” subset mainly consists of small faces and these cause difficulties for SFPD. However, this high drop in performance can be observed for almost all algorithms with double-digit frame rates. Especially, the detection of very small objects is difficult to implement with only few runtime losses. However, it was not the goal of our SFPD approach to perform very well with small faces, because it mainly targets close-range human–robot interaction scenarios. This was achieved by implementing the proposed network on a mobile robot and it is successfully used for real-time human–robot interaction in a demo application (see Figure 5).

In conclusion, SFPD achieves good results in both person and face detection. Furthermore, it was important to us that the two detection tasks are executed with high frame rate, so that additional modules such as face recognition can be integrated into the pipeline and still real-time processing of the entire system is guaranteed. This goal could be achieved with a frame rate of 40 fps for the detection of faces and persons. The main advantages of our SFPD are that it detects both faces and persons simultaneously and reaches high framerates with good detection performance for both tasks. Compared to all other models, SFPD is either faster or more reliable in terms of detection performance. Thus, SFPD represents the optimal network for real-time human–robot interaction applications.

### 4.4. Limitations

Although our SFPD network shows good results on the test datasets, especially in relation to the required computational time, there are still some limitations regarding the recognition performance.

SFPD has difficulties to detect very small objects. This can be explained by the fact that they often consist of only a few pixels and it is therefore difficult to extract meaningful features which are necessary for correct detection. This is particularly evident in the results on the WIDER Face validation “hard” subset which consists mainly of small faces. A possible solution approach would be to scale the input images for the network to a larger format so that the individual objects would comprise more pixels. However, this would have negative effects on the runtime of the CNN, which was no option due to the real-time requirements of the targeted human–robot interaction application.

Another difficulty of SFPD is that it has problems to separate several objects that are located close to each other, especially, in crowded scenes. For highly overlapping objects, this effect will be further intensified. An approach to solve this problem would be to lower the NMS threshold, so that, e.g., two strongly overlapping objects can be recognized as two objects and none is rejected because of a too high IoU between the boxes. However, this would result in multiple detections of the same objects.

Despite these limitations, we believe that the SFPD algorithm offers a good trade-off between detection performance and inference time making it a good detection framework for many real-world applications. In particular, it is suitable for human–robot interaction, which requires real-time processing and does not suffer from the limitations detecting very small faces and handling crowded scenes (due to close-range interaction with a quite small number of people).

Future work may address improving performance with low-quality images that may occur, e.g., due to bad lighting or low-cost camera hardware. This may be done by collecting additional low-quality training data or applying data augmentation that degrades the image quality.

## 5. Conclusions

Our newly developed SFPD approach is able to detect faces and persons simultaneously in real-time. For this purpose, it employs a joint CNN backbone with shared feature maps and separate detection layers for each task. The difficulty for training this network was the fact that available datasets only contain annotations of bounding box coordinates for one of the two detection tasks. By applying a special training procedure and by designing a custom multi-task loss function, this problem could be addressed during training and a completely end-to-end trainable framework was created. Thereby, SFPD does not need any auxiliary steps during training, such as pre-training individual network parts, splitting up the training process, freezing single network layers or creating additional annotations for datasets, as it is mostly the case with other multi-task learning networks. SFPD performs well against other algorithms. Person detection was evaluated on the Pascal VOC datasets and face detection on the WIDER Face dataset. Moreover, our approach is capable of processing 40 fps. It is superior to all other algorithms in at least one of processing speed, detection performance or providing both face and person detections. Because of the good trade-off between detection performance for both detection tasks and inference time, SFPD represents a useful framework especially for close-range human–robot interaction scenarios and many more real-world applications.

## Figures and Tables

**Table 1:** Results of our SFPD network and other detectors on the Pascal VOC test “person” subsets 2007 and 2012.

	VOC Test Set			
Method	2007	2012	fps	GPU
Fast R-CNN [16]	69.9	72.0	1	Tesla K40
Faster R-CNN [17]	76.7	79.6	5	Tesla K40
			7	Titan X
SSD300 [25]	76.2	79.4	46	Titan X
SSD512 [25]	79.7	83.3	19	Titan X
YOLO [22]	-	63.5	45	Titan X
YOLOv2 [23]	-	81.3	40	Titan X
EfficientDet-D2 [27] ^†^	78.8	81.9	43	Titan V
EfficientDet-D3 [27] ^†^	81.1	85.6	27	Titan V
RetinaNet [26]	78.3	-	14	Tesla V100
FoveaBox [29]	79.5	-	16	Tesla V100
SFPD [ours]	78.1	81.5	40 *	RTX 2080 Ti

All frameworks (except ^†^ denoted) were trained exclusively with person annotations from the Pascal VOC trainval sets of 2007 and 2012; the inference time was determined with a batch size of one; * at our SFPD method denotes that both faces and persons are detected within this inference time; ^†^ denotes that the network is trained on MS COCO [66] and not on Pascal VOC datasets.

**Table 2:** Results of our SFPD network and other detectors on the WIDER Face validation set.

	WIDER Validation Set				
Method	Easy	Medium	Hard	fps	GPU
YOLOv2 [23] (from [49])	33.1	29.3	13.8	40	Titan X
ACF-WIDER [67]	65.9	54.1	27.3	20	CPU
Two-stage CNN [7]	68.1	61.8	32.3	-	-
YOLOv3 [24] (from [49])	68.3	69.2	51.1	35	Titan X
Multi-scale Cascade CNN [7]	69.1	66.4	42.4	-	-
Faceness-WIDER [68]	71.3	63.4	45.6	-	-
LDCF+ [69]	79.0	76.9	52.2	3	CPU
YOLO-face (darknet-53) [49]	82.5	77.8	52.5	45	GTX 1080 Ti
Multitask Cascade CNN [40]	84.8	82.5	59.8	16	Titan Black
ScaleFace [45]	86.8	86.7	77.2	4	Titan X
YOLO-face (deeper darknet) [49]	89.9	87.2	69.3	38	GTX 1080 Ti
DSFD (ResNet50) [47]	93.7	92.2	81.8	22	Tesla P40
Face R-FCN [41]	94.7	93.5	87.4	3	Tesla K80
FCOS [28] (from [48])	95.0	90.6	55.0	-	-
FAN [43]	95.2	94.0	90.0	11	Titan Xp
FoveaBox [29] (from [48])	95.6	93.5	67.8	11	Tesla V100
DBCFace [48]	95.8	95.0	90.3	7	GTX 1080 Ti
FDNet [70]	95.9	94.5	87.9	-	-
PyramidBox [44]	96.1	95.0	88.9	3	Titan RTX
DSFD (ResNet152) [47]	96.6	95.7	90.4	-	-
RetinaFace [46]	96.9	96.1	91.8	13	Tesla P40
SFPD [ours]	80.5	73.6	51.3	40 *	RTX 2080 Ti

All frameworks were trained exclusively with face annotations from the WIDER Face train set; the inference time was determined with a batch size of one; * at our SFPD method denotes that both faces and persons are detected within this inference time.

## Data Availability

The WIDER Face dataset can be obtained at http://shuoyang1213.me/WIDERFACE/ (accessed on 3 August 2021). The Pascal VOC datasets can be obtained at http://host.robots.ox.ac.uk (accessed on 3 August 2021).

## Abbreviations

The following abbreviations are used in this manuscript:

CNN	convolutional neural network
DPM	deformable parts model
fps	frames per second
GIoU	generalized intersection over union
GPU	graphics processing unit
HCI	human-computer interaction
HOG	histogram of oriented gradient
IoU	intersection over union
*L*	loss
*L*${}_{conf}$	confidence loss
*L*${}_{reg}$	regression loss
MTL	multi-task learning
NMS	non-maximum suppression
ReLU	rectified linear unit
SFPD	simultaneous face and person detection
SGD	stochastic gradient descent
